# Lumican Expression in Diaphragm Induced by Mechanical Ventilation

**DOI:** 10.1371/journal.pone.0024692

**Published:** 2011-09-09

**Authors:** Li-Fu Li, Bao-Xiang Chen, Ying-Huang Tsai, Winston W.-Y. Kao, Cheng-Ta Yang, Pao-Hsien Chu

**Affiliations:** 1 Division of Pulmonary and Critical Care Medicine, Department of Internal Medicine, Chang Gung Memorial Hospital, Taipei, Taiwan; 2 Department of Medicine, College of Medicine, Chang Gung University, Taoyuan, Taiwan; 3 Graduate Institute of Clinical Medical Science, College of Medicine, Chang Gung University, Taoyuan, Taiwan; 4 Department of Respiratory Care, College of Medicine, Chang Gung University, Taoyuan, Taiwan; 5 Section of Respiratory Care, Department of Integrated Diagno-Therapeutics, National Taiwan University Hospital, Taipei, Taiwan; 6 Crawley Vision Research Center/Department of Ophthalmology, College of Medicine, University of Cincinnati, Cincinnati, Ohio, United States of America; 7 The First Cardiovascular Division, Department of Internal Medicine, Chang Gung Memorial Hospital, Taipei, Taiwan; University Hospital Freiburg, Germany

## Abstract

**Background:**

Diaphragmatic dysfunction found in the patients with acute lung injury required prolonged mechanical ventilation. Mechanical ventilation can induce production of inflammatory cytokines and excess deposition of extracellular matrix proteins via up-regulation of transforming growth factor (TGF)-β1. Lumican is known to participate in TGF-β1 signaling during wound healing. The mechanisms regulating interactions between mechanical ventilation and diaphragmatic injury are unclear. We hypothesized that diaphragmatic damage by short duration of mechanical stretch caused up-regulation of lumican that modulated TGF-β1 signaling.

**Methods:**

Male C57BL/6 mice, either wild-type or *lumican*-null, aged 3 months, weighing between 25 and 30 g, were exposed to normal tidal volume (10 ml/kg) or high tidal volume (30 ml/kg) mechanical ventilation with room air for 2 to 8 hours. Nonventilated mice served as control groups.

**Results:**

High tidal volume mechanical ventilation induced interfibrillar disassembly of diaphragmatic collagen fiber, lumican activation, type I and III procollagen, fibronectin, and α-smooth muscle actin (α-SMA) mRNA, production of free radical and TGF-β1 protein, and positive staining of lumican in diaphragmatic fiber. Mechanical ventilation of lumican deficient mice attenuated diaphragmatic injury, type I and III procollagen, fibronectin, and α-SMA mRNA, and production of free radical and TGF-β1 protein. No significant diaphragmatic injury was found in mice subjected to normal tidal volume mechanical ventilation.

**Conclusion:**

Our data showed that high tidal volume mechanical ventilation induced TGF-β1 production, TGF-β1-inducible genes, e.g., collagen, and diaphragmatic dysfunction through activation of the lumican.

## Introduction

Acute lung injury (ALI) and its most severe manifestation, acute respiratory distress syndrome (ARDS), are inhomogeneous lung diseases characterized by the initial diffuse inflammatory reactions, neutrophil influx into the lungs, loss of epithelial and endothelial integrity, the development of noncardiogenic pulmonary edema and is followed by fibroblast proliferation and extracellular matrix accumulation [1]–[3]. The prognosis is poor and often results in the need for long-term support of mechanical ventilation due to deficits of diaphragmatic force and endurance [4]. Mechanical ventilation has been shown to increase diaphragmatic injury (VIDD: ventilator-induced diaphragmatic dysfunction) associated with the increase of protein oxidation and inflammatory cytokines such as macrophage inflammatory protein-2 (MIP-2), interferon (IFN) γ-inducible protein of 10 kD (IP-10), and transforming growth factor (TGF)-β1 [5]–[7]. The use of high tidal volume (V_T_) in normal animals mimics this overdistention of the normal lung. Previous studies of human dermal fibroblasts has shown that TGF-β1 caused a marked increase of the production of type I and III collagens, fibronectin, and α-smooth muscle actin (α-SMA) [8]. Besides implicated in the collagen formation in the fibroproliferative phase of ARDS, TGF-β1 may play an important role in the early phase of ARDS [9].

Lumican belongs to the family of small leucine-rich repeat proteoglycans (SLRPs) binding collagen fibrils and are important in regulating collagen fibrillogenesis, i.e., fibril diameter and interfibrillar spacings [10]–[11]. The expression of lumican may also have proinflammatory effects including the interactions with MIP-2, TGF-β1, extracellular signal regulated kinases (ERK) 1/2, and toll-like receptors [11]–[12]. Lumican deficient mice exhibit corneal opacity as well as skin and tendon fragility associated with disorganized and loosely packed collagen fibers [13]–[14]. We hypothesized that short duration of mechanical stretch augmented diaphragmatic damage, and production of TGF-β1 via lumican pathway. In high tidal volume ventilation-induced diaphragmatic injury model in mice, we examined the relationships between different tidal volume of mechanical ventilation, TGF-β1-inducible genes, and TGF-β1 production using the lumican deficient mice.

## Results

### Physiologic data

No statistical difference was found in pH, PaO2, PaCO2, mean arterial pressure, and peak inspiratory pressure at the beginning versus the end of 8 hours of mechanical ventilation (Table 1). The normovolemic statuses of mice were maintained by monitoring mean artery pressure.

**Table 1:** Physiologic conditions at the beginning and end of ventilation.

	Control nonventilated wild-type	Control nonventilated lum^−/−^	V_T_ 10 ml/kg wild-type	V_T_ 30 ml/kg wild-type	V_T_ 30 ml/kg lum^−/−^
PH	7.42±0.05	7.40±0.04	7.37±0.04	7.35±0.07	7.36±0.05
PaO_2_ (mmHg)	98.1±0.3	97.9±0.2	90.7±1.1	91.8±1.3	92.1±0.9
PaCO_2_ (mmHg)	39.2±0.1	38.9±0.2	37.5±0.7	34.1±1.4	35.2±1.8
MAP (mmHg)					
Start	83±1.5	84±1.2	83.1±1.3	82.2±1.9	82.5±1.7
End	82±0.6	82±0.5	79.8±2.7	75.6±5.3	76.1±3.6
PIP, mm Hg					
Start			15.1±1.3	23.5±1.7	23.2±1.5
End			16.7±2.1	27.1±3.5	26.9±2.4

Arterial blood gases and mean arterial pressure were obtained from nonventilated mice and mice ventilated at a tidal volume of 10 ml/kg or 30 ml/kg for 8 hours (n = 10 per group). *Lumican*-null mice = lum^−/−^; MAP = mean arterial pressure; PIP: peak inspiratory pressure; V_T_ = tidal volume. The physiological data of control groups were similar during the experiment and were used as the beginning data of ventilation.

### Aggravated disarray of fibers after ventilation-induced injury in the diaphragm

**Figure 1:**
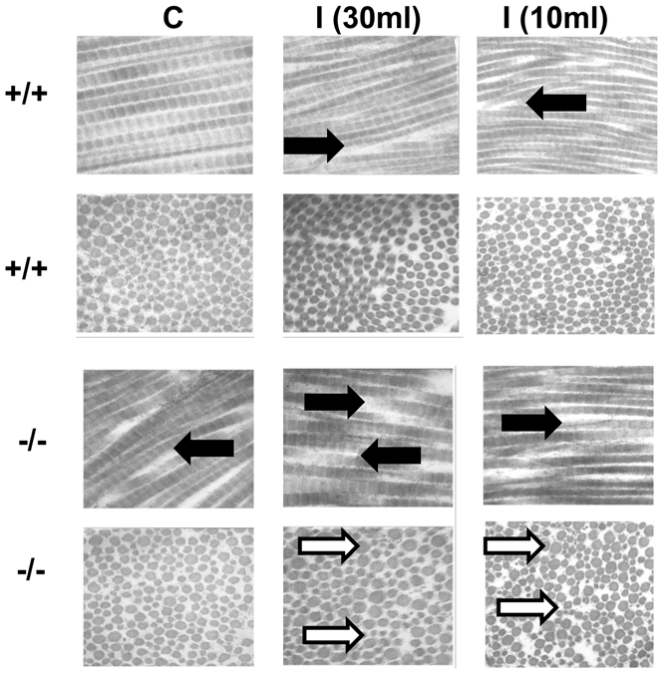
Electron microscopy of the diaphragm. Representative micrographs (x80,000, longitudinal section and transverse section) of the diaphragmatic sections were from control nonventilated mice and mice ventilated at tidal volume 10 ml/kg or 30 ml/kg for 8 hours with room air (n = 2 per group). Increasing of interfibrillar spacings and disrupted diaphragmatic collagen fibers (black arrow) after ventilation injury, and variation of the cross-section were identified (white arrows). The severity were more aggravated in the *lumican*-null mice. Scale bars represent 115 nm. (+/+, wild type mice; −/−, *lumican*-null mice; C, control nonventilated; **I, 10ml**, ventilated at tidal volume 10 ml/kg; **I, 30ml**, ventilated at tidal volume 30 ml/kg)

Electromicroscpy was used to determine the effects of mechanical ventilation on the ultrastructures of diaphragmatic collagen fibers. Disruptions of diaphragmatic collagen fiber and increase of interfibrillar spacing were observed in mice subjected to high tidal volume mechanical ventilation for 8 hours as compared to that of control, nonventilated mice (Figure 1). In the absence of lumican of *lumican*-null mice, there was a reduction of damage of diaphragmatic fiber. This suggested that lumican may be involved in the pathogenesis of ventilator-induced diaphragmatic injury. No significant disruptions of diaphragmatic collagen fiber and increase of interfibrillar spacing were observed in mice subjected to normal tidal volume mechanical ventilation (10 ml/kg) for 8 hours as compared to that of the control, non-ventilated mice.

**Figure 2:**
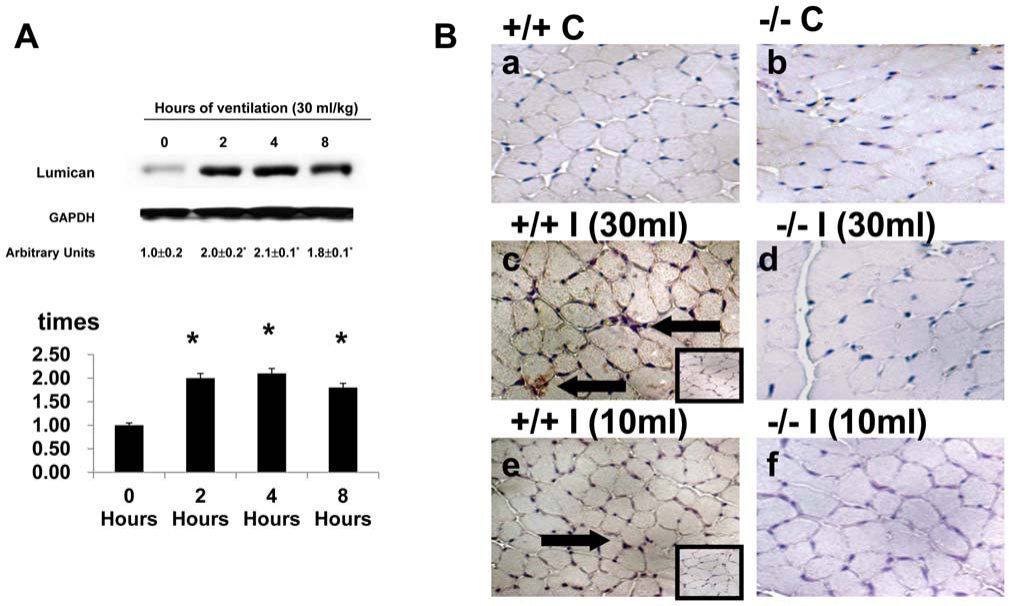
Lumican deficient mice reduced high tidal volume ventilation-induced lumican activation in diaphragm. (**A**) The mice were ventilated at 30 ml/kg for 2, 4, and 8 hours with room air. Western blot was performed using an antibody, which recognizes the lumican expression (Top Panel) and an antibody that recognizes glyceraldehydes-phosphate dehydrogenase (GAPDH) expression (Middle Panel). Arbitrary units were expressed as the ratio of lumican to GAPDH (Bottom Panel) (n = 3–5 per group). *P<0.05 versus control, nonventilated mice. (**B**) Representative photomicrographs (x400) with lumican staining of paraffin diaphragm sections with immunohistochemistry were from control, nonventilated mice and mice ventilated at 10 ml/kg or 30 ml/kg for 8 hours with room air. (n = 3–5 per group). An inset panel showed staining using isotype-matched controls. Positive dark brown diaminobenzidine (DAB) staining of membranes of muscle fiber is identified by arrows. (+/+, wild type; −/−, lum^−/−^;C, control nonventilated; I, ventilated injury)

To determine the time courses of stretch-induced lumican activation, we measured level of lumican in diaphragm at 2 to 8 hours of mechanical ventilation. The level of lumican increased after 2 hours of mechanical ventilation and remained at high levels after 8 hours of mechanical ventilation as compared to that of control, nonventilated mice (Figure 2A). However, the level of lumican increased after 2 hours of normal tidal volume mechanical ventilation (10 ml/kg) but decreased after 4 and 8 hours of mechanical ventilation as compared to that of the control, nonventilated mice (arbitrary units of lumican: Control = 1.0±0.1, V_T_ 10 ml/kg, 2 h = 1.3±0.3^*^, V_T_ 30 ml/kg, 4 h = 1.1±0.4; V_T_ 10 ml/kg, 8 h = 1.0±0.2, ^*^P<0.05 versus Control). With immunohistochemistry, we then further examined the role of lumican up-regulation and determined the cells types involved in the ventilation-induced diaphragmatic injury (Figure 2B). The positive staining of lumican in diaphragm fiber of mice ventilated at tidal volume (V_T_) 30 ml/kg increased as compared to control, nonventilated mice, and that of lumican null mice ventilated at V_T_ 30 ml/kg. However, no significant increase of staining of lumican was found in mice ventilated at V_T_ 10 ml/kg. Nonimmune rabbit immunoglobulin G control showed no detectable staining (Figure 2B).

### Inhibition of lumican activation with lumican deficient mice reduced ventilation-induced TGF-β1 production, and type I and type III procollagen, fibronection and α-SMA mRNA expression, and malondialdehyde (MDA) production

**Figure 3:**
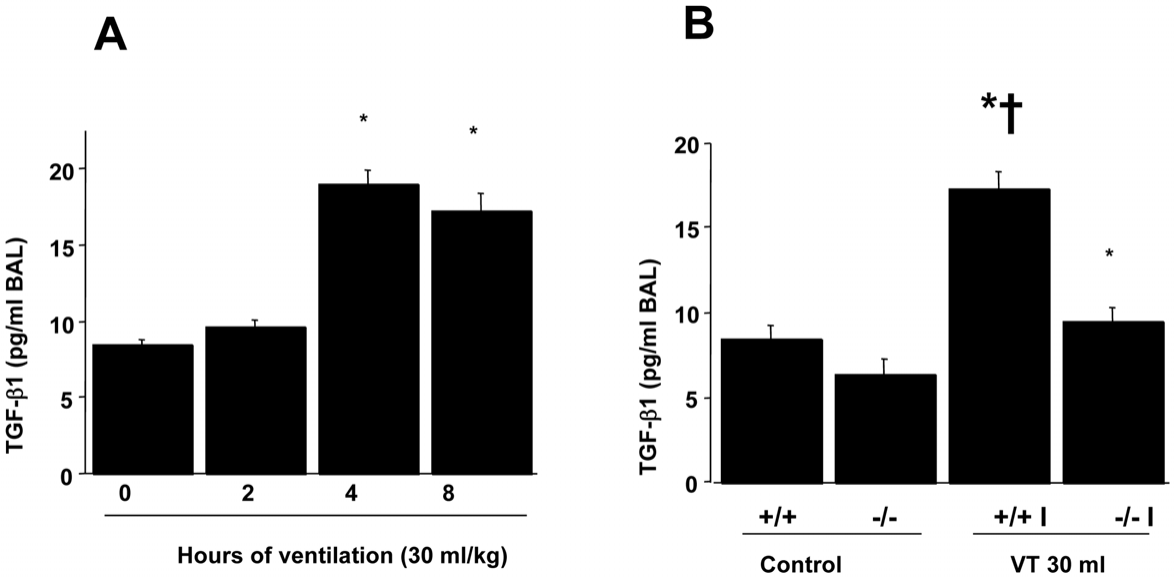
Lumican deficient mice reduced high tidal volume ventilation-induced transforming growth factor-β1 (TGF-β1) production. TGF-β1 production in bronchoalveolar lavage (BAL) fluid was from control, nonventilated mice and mice ventilated at V_T_ 30 ml/kg for 2, 4, and 8 hours (**A**, n = 5 per group) or V_T_ 30 ml/kg for 8 hours (**B**, n = 5 per group). * *P*<0.05 versus control, nonventilated mice; †*P*<0.05 versus lum^−/−^ mice. WT: wild type C57BL/6 mice. (+/+, wild type; −/−, lum^−/−^; C, control nonventilated; I, ventilated injury)

**Figure 4:**
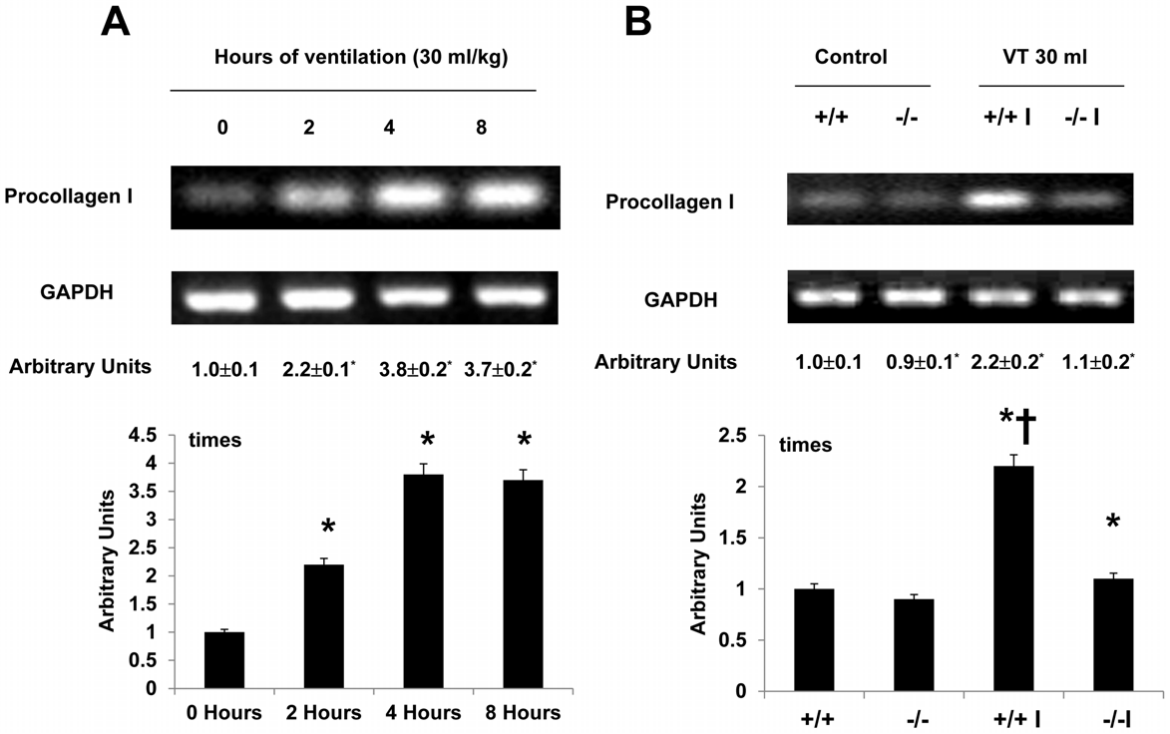
Lumican deficient mice reduced high tidal volume ventilation-induced type I procollagen mRNA expression in diaphragm. The mice were ventilated at V_T_ 30 ml/kg at indicated time periods (**A**, n = 5 per group) or V_T_ 30 ml/kg for 2 hours (**B**, n = 5 per group). Reverse transcription-polymerase chain reaction (RT-PCR) was performed for type I procollagen mRNA (Top Panel), GAPDH mRNA (Middle Panel), and arbitrary units (Bottom Panel). Arbitrary units were expressed as the ratios of type I procollagen mRNA to GAPDH. *P<0.05 versus control, nonventilated mice; †*P*<0.05 versus lum^−/−^ mice. WT: wild type C57BL/6 mice. (+/+, wild type; −/−, lum^−/−^; C, control nonventilated; I, ventilated injury)

To explore the chemoattractants associated with ventilator-induced diaphragmatic injury, we measured TGF-β1 (Figure 3). The production of TGF-β1 increased at 2 hours of mechanical ventilation and remained 2-fold elevated for up to 8 hours as compared to that of control, nonventilated mice. However, the production of TGF-β1 increased at 2 hours and 4 hours of normal tidal volume mechanical ventilation (10 ml/kg) but decreased after 8 hours of mechanical ventilation as compared to that of the control, nonventilated mice. The increases of TGF-β1 protein production were significantly lowered in lumican deficient mice (Control = 8.5±1.1 pg/ml, V_T_ 10 ml/kg, 2 h = 14.6±1.4 pg/ml ^*^, V_T_ 30 ml/kg, 4 h = 16.5±1.8 pg/ml ^*^; V_T_ 10 ml/kg, 8 h = 9.2±1.5 pg/ml, V_T_ 10 ml/kg, lum-null = 9.5±1.2 pg/ml, P<0.05 versus Control). To determine if the increase of mechanical ventilation-induced diaphragmatic damage was accompanied by up-regulation of procollagens, we measured type I and type III procollagen, fibronectin, and α-SMA mRNA (Figure 4–7). There were time-dependent increases in TGF-β1 protein production, expressions of type I and type III procollagen, fibronectin, and α-SMA mRNA in the V_T_ 30 ml/kg mice as compared to those of control, nonventilated mice. In contrast, the increases of TGF-β1 protein production and expressions of type I and type III procollagen, fibronectin, and α-SMA mRNA were significantly lowered in lumican deficient mice. Since increase of free radical has been found to associate with stretch-induced diaphragmatic injury, thus MDA assay was performed to elucidate possible effects of free radical on the pathogenesis caused by ventilation [6]. The MDA activity of diaphragm was increased in mice ventilated at V_T_ 30 ml/kg as compared to that of control, nonventilated mice and the effect was reduced with lumican deficient mice (Figure 8).

**Figure 5 pone-0024692-g005:**
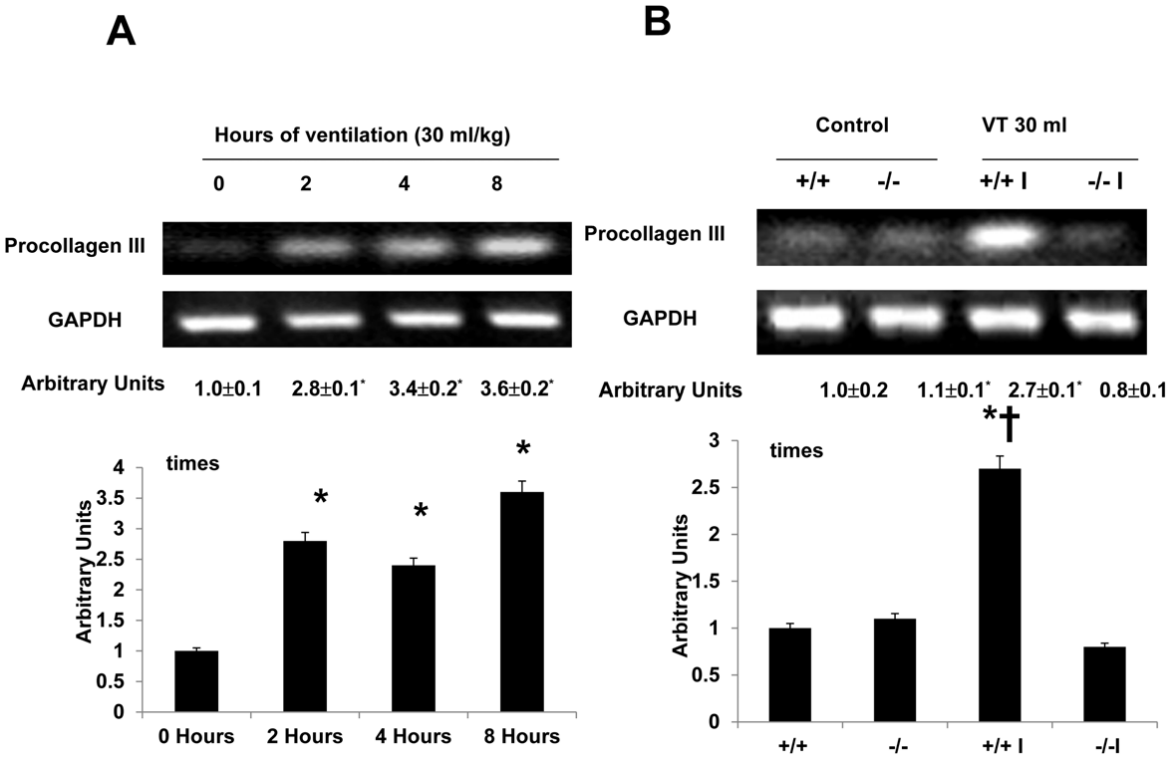
Lumican deficient mice reduced high tidal volume ventilation-induced type III procollagen mRNA expression in diaphragm. The mice were ventilated at V_T_ 30 ml/kg at indicated time periods (**A**, n = 5 per group) or V_T_ 30 ml/kg for 2 hours (**B**, n = 5 per group). RT-PCR was performed for type III procollagen mRNA (Top Panel), GAPDH mRNA (Middle Panel), and arbitrary units (Bottom Panel). Arbitrary units were expressed as the ratios of type III procollagen mRNA to GAPDH. *P<0.05 versus control, nonventilated mice; †*P*<0.05 versus lum^−/−^ mice. WT: wild type C57BL/6 mice. (+/+, wild type; −/−, lum^−/−^; C, control nonventilated; I, ventilated injury)

**Figure 6 pone-0024692-g006:**
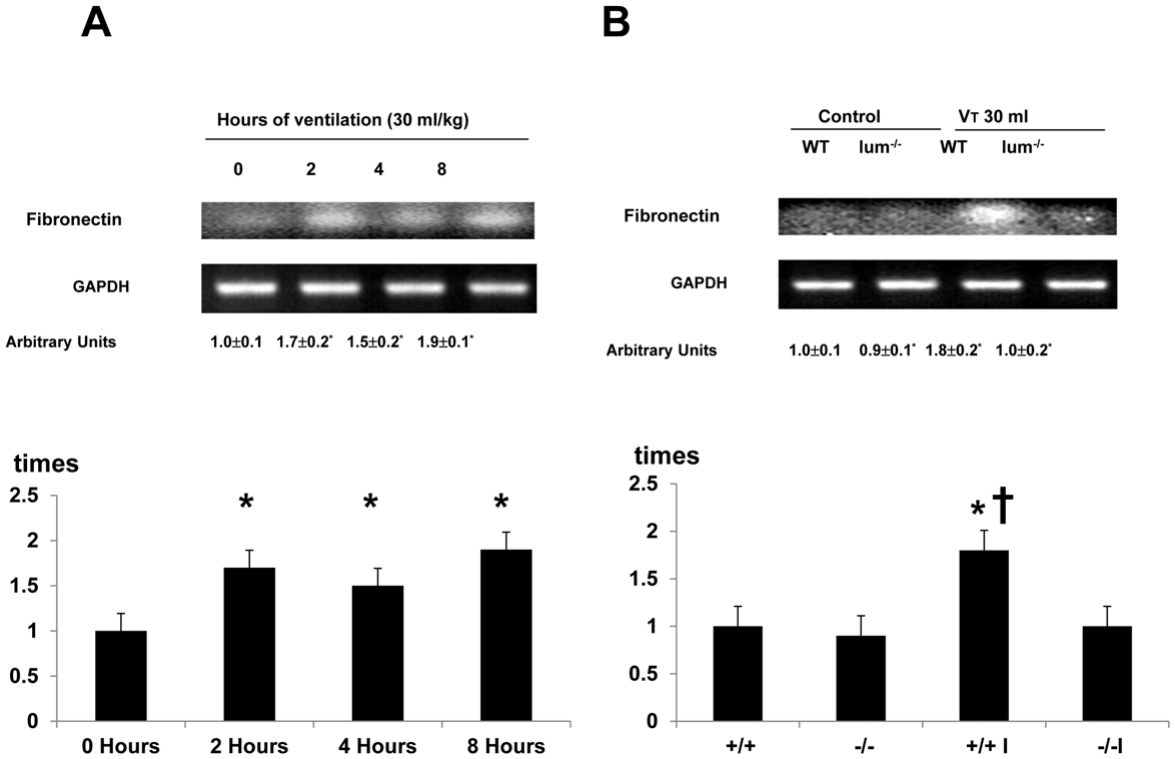
Lumican deficient mice reduced high tidal volume ventilation-induced fibronectin mRNA expression in diaphragm. The mice were ventilated at V_T_ 30 ml/kg at indicated time periods (**A**, n = 5 per group) or V_T_ 30 ml/kg for 2 hours (**B**, n = 5 per group). RT-PCR was performed for fibronectin mRNA (Top Panel), GAPDH mRNA (Middle Panel), and arbitrary units (Bottom Panel). Arbitrary units were expressed as the ratios of fibronectin mRNA to GAPDH. *P<0.05 versus control, nonventilated mice; **†** *P*<0.05 versus lum^−/−^ mice. WT: wild type C57BL/6 mice.(+/+, wild type; −/−, lum^−/−^; C, control nonventilated; I, ventilated injury)

**Figure 7:**
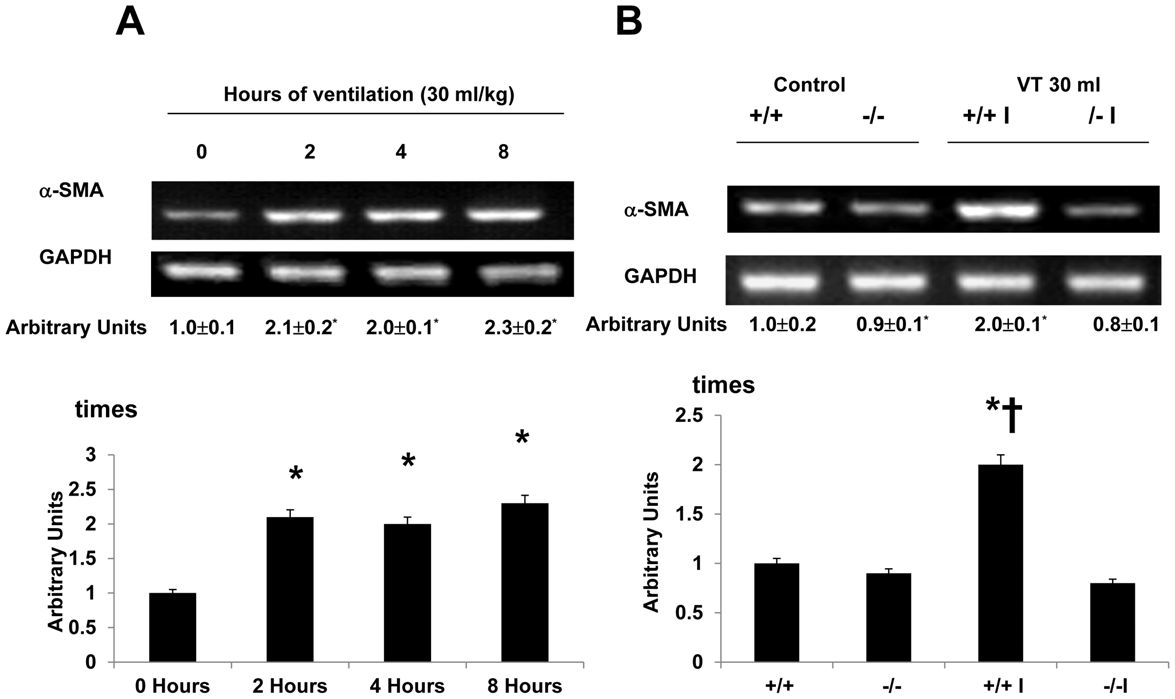
Lumican deficient mice reduced high tidal volume ventilation-induced α-SMA mRNA expression in diaphragm. The mice were ventilated at V_T_ 30 ml/kg at indicated time periods (**A**, n = 5 per group) or V_T_ 30 ml/kg for 2 hours (**B**, n = 5 per group). RT-PCR was performed for α-SMA mRNA (Top Panel), GAPDH mRNA (Middle Panel), and arbitrary units (Bottom Panel). Arbitrary units were expressed as the ratios of α-SMA mRNA to GAPDH. *P<0.05 versus control, nonventilated mice; **†** *P*<0.05 versus lum^−/−^ mice. α-SMA, α-smooth muscle actin; WT: wild type C57BL/6 mice. (+/+, wild type; −/−, lum^−/−^; C, control nonventilated; I, ventilated injury)

**Figure 8:**
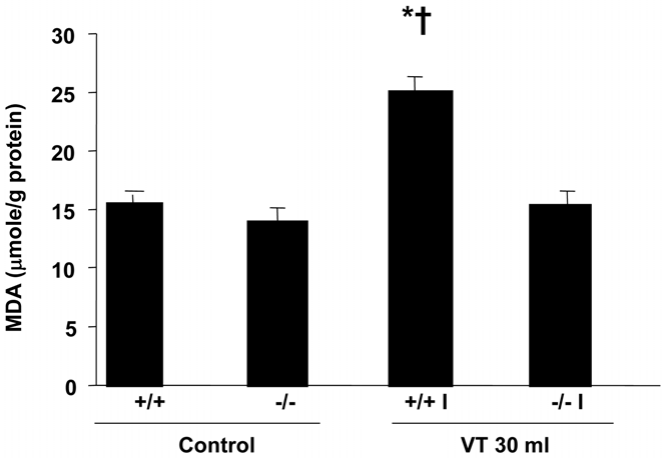
Lumican deficient mice reduced high tidal volume ventilation-induced malondialdehyde (MDA) production in diaphragm. The mice were ventilated at V_T_ 30 ml/kg for 8 hours (n = 5 per group). * *P* <0.05 versus control, nonventilated mice; **†** *P*<0.05 versus lum^−/−^ mice. WT: wild type C57BL/6 mice. (+/+, wild type; −/−, lum^−/−^; C, control nonventilated; I, ventilated injury)

## Discussion

Recent studies suggested that increased inflammatory cytokines, extracellular matrix, and collagen formation might occur in the first week of ARDS, which caused reduced pulmonary compliance and severe hypoxemia. Identification of the mechanisms regulating fibrogenesis of ARDS will help development of better treatment regimens for diaphragmatic and lung injury in ARDS patients. In this injurious mechanical ventilation model of mouse, we found that high tidal volume ventilation increased interfibillar spacings and disruptions of diaphragmatic collagen, production of TGF-β1, TGF-β1-indubible genes, and free radical. Our hypothesis is lumican pathway was regulated by TGF-ß expression in the diaphragmatic injury.

Previous studies have shown that patients experienced difficulty in weaning from prolonged mechanical ventilation may be linked to diaphragm dysfunction due to abnormal fiber remodeling resulting from oxidative stress, and repair of structural injury [6], [15]. The onset of VIDD is rapid within 6 hours after the initiation of mechanical ventilation and the magnitude of impairment of diaphragmatic contraction increased with time on the ventilator [4]. We found that interfibrillar disassembly of diaphragmatic collagen fiber and oxidative injury after 8 hours of mechanical ventilation. We then explored the major physiologic trigger leading to these alterations.

Collagen, as a supportive structure in skeletal muscle and tendon is the most abundant protein of the extracellular matrix [16]. Mechanical ventilation for 2 to 5 hours in rats have been shown to increase the expression of type III procollagen, the first collagen type involved in the remodeling in the evolution of fibrogenesis and up-regulation of hyaluronan (HA) synthase 3 mRNA and HA production by fibroblasts, contributing to extracellular matrix-induced inflammatory changes involved in ventilator-induced lung injury (VILI) [17], [18]. We found that mechanical ventilation increased expressions of mesenchymal markers, including type I and type III procollagen, fibronectin, and α-SMA mRNA in a time-dependent manner. TGF-β1 is a multifunctional cytokine that plays an important role in the induction of extracellular matrix deposition by fibroblasts and may induce cytoskeletal reorganization found in epithelial-mesenchymal transition [19], [20]. Using human dermal fibroblasts, others showed that TGF-β1 increased the production of types I and III collagens and fibronectin but the chemotactic effects of TGFβ1 have been shown to occur at concentrations much lower than those required for extracellular matrix induction in the lung [8], [20]. TGFβ1 is no longer chemotactic at higher concentrations and may attract cells toward its source of delivery [20]. As a major pro-fibrogenic cytokine, TGF-β1 was also found in the pathogenesis of acute lung injury related with mechanical ventilation and oxygen injury [9], [21]. Mechanical ventilation-induced oxidative stress is an important factor regulating mechanical ventilation-induced diaphragmatic contractile dysfunction and is a potent stimulus for the production of TGF-β1 [22]. We found that mechanical ventilation resulted in increases of TGF-β1 and free radical (malondialdehyde) production.

Lumican is present in a variety of non-corneal tissues, *e.g.* cartilage, heart, lung, skin, kidney, and skeletal muscle, as a smaller, more homogeneous, poorly sulfated or nonsulfated glycoprotein [10]. Mouse lumican is a 338-amino acid protein with high sequence homology to bovine, human, and chicken lumican [10]. Experimental acute lung injury model of rats showed that fragmented proteoglycans increased with high tidal volume ventilation may bind to the surface of collagen fibrils and affect the collagen matrix assembly in connective tissues [23]. The increased production of proteoglycans are important in the transmission of stress between the extracellular matrix and may bind to different growth factors, such as TGF-β1 and fibroblast growth factor [12]. We found that up-regulation of lumican by ventilation was time-dependent. Using lumican deficient mice, we found decrease of disruptions of diaphragmatic collagen fiber, reduced TGF-β1 production, and subsequent expression of TGF-β1-inducible fibrotic genes, suggesting the involvement of lumican in the regulation of VIDD. However, the decrease of lumican expression after 8 hours of mechanical ventilation suggested that the lumican signal was only one of the many pathways contributing to diaphragmatic injury.

It is reasonable to speculate that no one single factor is solely responsible for lung fibrosis, rather a concerted expression of various factors and cytokines may account for the pathology seen in lung injury. For example, altered balance between angiogenic (MIP-2, plasminogen activator inhibitor-1, TGF-β1, and CXCL12) and angiostatic (IP-10 and CXCL11) chemokines may promote aberrant angiogenesis/fibrosis [19]. In a study of mechanical ventilation in brain-dead patients, others showed that there is no evidence of increased diaphragmatic inflammatory cell infiltration [24]. The injurious effects of remote organ systems on skeletal muscle may be mediated by the systemic transmission of oxidative stress via radical-inducing substances such as inflammatory cytokines [25]. We found that mechanical ventilation increased the level of TGF-β1 in bronchoalveolar fluid and free radical production in the diaphragm. Previous studies of mechanical ventilation in rats showed that increases of caspase-3 mediated myonuclear apoptosis, and excess proteoglycans such as biglycan, and metalloproteinases had been observed after mechanical ventilation [22], [23], [26]. We found that mechanical ventilation increased proteoglycans of lumican in diaphragm of mouse, which was associated with the activation of TGF-β1 and collagens. The expression of α-SMA, a marker of myofibroblasts, indicated the presence of an ongoing angiogenetic program determining mesenchymal phenotype. The predominant cell types involved in pulmonary fibrosis are fibroblasts and myofibroblasts, and the damaged epithelium can activate transformation of fibroblasts to myofibroblasts, epithelial-mesenchymal transition (EMT), through the secretion of TGF-β1 [14], [19], [20]. Similar to Levine's study in the diaphragms of ventilated humans [24], we did not find an increase of diaphragmatic inflammatory cells. Further experiments on lung injury may explore more about the relationship between TGF-β1 and EMT.

Though the ARDS network trial demonstrated that low is safer than high tidal volume ventilation, these findings have been questioned and the mechanisms of injury and protection need to be further examined [27]. The National Heart, Lung and Blood Institute working group on ARDS identified examination of the biology of stress-induced injury to the lung in health and disease as a fertile area of future research, because ventilation-induced release of cytokines may lead to systemic translocation and multisystem organ failure [28]. High tidal volumes in normal animals have been used to mimic overdistention of the less injured and thus more compliant areas of lung found in ARDS patients. These animal models have shown that simply over overdistending lung tissue, in the absence of acid aspiration or bacterial lipopolysaccharide causes production of cytokines and chemokines [29], [30]. Using an *in vivo* mouse model, we have demonstrated that high tidal volume mechanical ventilation induces diaphragmatic dysfunction, which are associated with activation of type I and III procollagen, fibronectin, and α-SMA mRNA, TGF-β1 and free radical production. This process was dependent, at least in part, on the lumican pathway. Our data add to the understanding of the effects of mechanical forces in the diaphragmatic injury in the lumican-null mice. In patients with ARDS in the early fibroproliferative phase, the inhibition of lumican may offer new treatment options for these patients.

### Materials and Methods

All procedures for handling mice were conformed to ARVO (Association of Research for Vision and Ophthalmology). Statements for the use of animals in research are approved by the Institutional Animal Care and Use Committee of Chang Gung Memorial Hospital (Permit Number: 2008090102). All surgery was performed under ketamine and xylazine anesthesia, and all efforts were made to minimize suffering.

### Generation and maintenance of lumican deficient (lumican^−/−^) Mice

Male C57BL/6, either wild-type or *lumican*-null (*Lum* ^−/−^) mice 3 months old, weighing between 25 and 30 g, were used. Lumican^−/−^ mice line, were generated by targeted gene disruption as previously described [31]. Briefly, a germ-line chimeric mouse, generated by blastocyst injection of a targeted embryonic stem cell clone from mouse strain J129/sv, was mated with C57BL/6 mice. The offspring were identified by polymerase chain reaction and southern hybridization. All animals used in this study (lumican^−/−^ and their wild-type littermates) were of a C57BL/6 genetic background and were identified by polymerase chain reaction. Mice that are homozygous for the targeted mutation are viable and fertile. The lumican deficient mice have serious functional defects including corneal opacity and fragile skin and tendon associated with disorganized and loosely packed collagen fibers. This proteoglycan has also been shown to participate in the regulation of many cellular functions including cell proliferation, migration, adhesion, and gene expression [32]. The lower expressions of the lumican protein in lumican^−/−^ mice were confirmed using Western blot analysis.

### Experimental groups

Animals were randomly distributed into six groups in each experiment: group 1, control, nonventilated wild-type mice (n = 2 for electromicroscopy; n = 5 each for western blot, type I and III procollagen, fibronectin, and α-SMA mRNA, malondialdehyde, immunohistochemistry, and TGF-β1); group 2, control, nonventilated lumican^−/−^ mice (n = 2 for electron microscopy; n = 5 each for western blot analysis, type I and III procollagen, fibronectin, and α-SMA mRNA, malondialdehyde, immunohistochemistry, and TGF-β1); group 3, V_T_ 30 ml/kg wild-type mice (n = 2 for electron microscopy; n = 5 for western blot, type I and III procollagen, fibronectin, and α-SMA mRNA at 2, 4, and 8 hours; n = 5 each for malondialdehyde, immunohistochemistry and TGF-β1); group 4, V_T_ 30 ml/kg lumican^−/−^ mice (n = 2 for electromicroscopy; n = 5 each for western blot, type I and III procollagen, fibronectin, and α-SMA mRNA, malondialdehyde, immunohistochemistry, and TGF-β1); group 5, VT 10 ml/kg wild-type mice (n = 2 for electron microscopy; n = 3 for immunohistochemistry; n = 3 for western blot and TGF-β1at 2, 4, and 8 hours) ; group 6, VT 10 ml/kg lumican^−/−^ mice (n = 3 each for western blot, immunohistochemistry, and TGF-β1).

### Ventilator protocol

We used an established mouse model of ventilator-induced lung injury (VILI), as previously described [33]. A 20-gauge angiocatheter was introduced into the tracheotomy orifice of mice under general anesthesia with intraperitoneal administration of ketamine (90 mg/kg) and xylazine (10 mg/kg). The mice were placed in a supine position on a heating blanket and then attached to a Harvard apparatus ventilator, model 55–7058 (Harvard Apparatus, Holliston, MA), set to deliver 10 ml/kg or 30 ml/kg at a rate of 65 breaths per minute for 2, 4, and 8 hours while breathing room air with zero end-expiratory pressure. The mice then received 0.9% saline containing maintenance ketamine (0.1 mg/g/h) and xylazine (0.01 mg/g/h) at a rate of 0.09 ml/10 g/h by a continuous intraperitoneal fluid pump. The tidal volume delivered by the ventilator was checked by fluid displacement from an inverted calibration cylinder. Continuous monitoring of end-tidal CO2 by a microcapnograph (Columbus Instruments, Columbus, OH) was performed, and respiratory frequencies of 65 breaths per minute for 10 ml/kg and 30 ml/kg were chosen in the experiment, with end-tidal CO2 at 30 to 40 mm Hg. Airway peak inspiratory pressure was measured with a pressure-transducer amplifier (Gould Instrument Systems, Valley View, OH) connected to the tubing at the proximal end of the tracheostomy. Mean arterial pressure was monitored every hour during mechanical ventilation using the same pressure-transducer amplifier connected to a 0.61-mm outer diameter (0.28-mm inner diameter) polyethylene catheter ending in the common carotid artery. At the end of the study period, heparinized blood was taken from the arterial line for analysis of arterial blood gas, and the mice were sacrificed. Control, nonventilated mice were anesthetized and sacrificed immediately.

### Transmission electon microscopy assay

The diaphragms were fixed in 3% glutaraldehyde in 0.1 M cacodylate buffer, (pH 7.4) for 1 hour at 4°C. The diaphragms were then postfixed in 1% osmium tetroxide (pH 7.4), dehydrated in a graded series of ethanol, and embedded in EPON-812. Thin sections (70 nm) were cut, stained with uranyl acetate and lead citrate, and examined on a Hitachi H-7500 EM transmission electron microscope.

### Western Immunoblot analysis

The diaphragms were homogenized in 0.5 ml of lysis buffer (20 mM HEPES pH 7.4, 1% Triton X-100, 10% glycerol, 2 mM ethylene glycol-bis (β-aminoethyl ether)-N, N, N′, N′-tetraacetic acid, 50 µM β-glycerophosphate, 1 mM sodium orthovanadate, 1 mM dithiotreitol, 400 µM aprotinin, and 400 µM phenylmethylsulfonyl fluoride), transferred to eppendorff tubes and placed on ice for 15 min. Tubes were centrifuged at 14,000 rpm for 10 min at 4°C and supernatant was flash frozen. Same amount of proteins from crude cell lysates determined by the Bradford method were resolved by 10% bis-acrylamide gel electrophoresis and electrotransferred to Immobilon-P membranes (Millipore Corp., Bedford, MA, USA). For assay of lumican and glyceraldehydes-phosphate dehydrogenase (GAPDH) protein expression, immunoanalyses were performed with antibodies against lumican and GAPDH (Santa Cruz Biotechnology, Santa Cruz, CA, USA). Blots were developed by enhanced chemiluminescence (NEN Life Science Products, Boston, MA, USA).

### Reverse transcription-polymerase chain reaction (RT-PCR)

For isolating total RNA, the diaphragms were homogenized in TRIzol reagents (Invitrogen Corporation, Carlsbad, CA) according to the manufacturer's instructions. Total RNA (1 µg) was reverse transcribed by using a GeneAmp PCR system 9600 (PerkinElmer, Life Sciences, Inc., Boston, MA), as previously described [34]. The following primers were used for PCR: type I procollagen, forward primer 5′-TGTGCCACTCTGACTGGAAGA-3′ and reverse primer 5′-CAGACGGCTGAGTAGGGAACA-3′; type III procollagen, forward primer 5′-GGAAAGGATGGAGAGTCAGGAA-3′ and reverse primer 5′-CATTGCGTCCATCAAAGCCT-3′; fibronectin: forward primer 5′-CGCTGTGACAACTGCCGTA-3′and reverse primer 5′-TTGTAGTTGTGGCCGGTGG -3′; α-SMA: forward primer 5′-GAACCCTGAGACGCTGCTCCAGCTATGTG-3′ and reverse primer 5′-CAGTAGTCACGAAGGAATAGCCACGC-3′, and GAPDH as internal control by using the following primers: forward primer 5′-AATGCATCCTGCA CCACCAA-3′ and reverse primer 5′-GTAGCCATATTCATTGTCATA-3′ (Integrated DNA Technologies, Inc., Coralville, IA) [35].

### Measurement of malondialdehyde

The diaphragms were homogenized in phosphate buffered saline containing butylated hydroxytoluene. The malondialdehyde in the protein extracts was measured using the Oxiselect TBARS assay kit (Cell Biolabs, San Diego, CA) containing thiobarbituric acid reactive substances. Each sample was run in duplicate and expressed as µmole/g protein according to the manufacturer's instructions.

### Measurement of TGF-β1

At the end of the study period, the lungs were lavaged via tracheostomy with a 20-gauge angiocatheter (sham instillation) 3 times with 0.6 ml of 0.9% normal saline. The effluents were pooled and centrifuged at 2,000 rpm for 10 minutes. Supernatants were frozen at −80°C for further analysis of the cytokine. TGF-β1 with a lower detection limit of 4.61 pg/ml was activated from the latent form and measured in BAL fluid by using a commercially available immunoassay kit containing primary polyclonal anti-mouse TGF-β1 antibodies (Biosource International, Camarillo, CA, USA). Each sample was run in duplicate according to the manufacturer's instructions.

### Immunohistochemistry

The diaphragms from control, nonventilated mice, and ventilated mice exposed to 10 ml/kg or 30 ml/kg ventilation for 8 hours while breathing room air were paraffin embedded, sliced at 4 µm, deparaffinized, antigen unmasked in 10 mM sodium citrate (pH 6.0), incubated with normal rabbit IgG or anti-lumican primary antibody (1∶100), and biotinylated goat anti-rabbit secondary antibody (1∶100) according to the manufacturer's instruction for an immunohistochemical kit (Santa Cruz Biotechnology, Santa Cruz, CA). The specimens were further incubated with horseradish peroxidase-streptoavidin complex, the immune reaction was visualized with diaminobenzidine (DAB), and counterstained by hematoxylin. A dark-brown DAB signal, identified by arrows, indicated positive staining of lumican of skeletal muscle cells, whereas shades of light blue signified nonreactive cells.

### Statistical evaluation

The type I procollagen, type III procollagen, fibronectin, and α-SMA mRNA and Western blots were quantitated by using a National Institutes of Health (NIH) image analyzer, ImageJ 1.27z (National Institute of Health, Bethesda, MD) and presented as arbitrary units. Values were expressed as the mean ± SD for at least 5 separate experiments. The MDA and TGF-β1 were conducted by using Statview 5.0 (Abascus Concepts Inc. Cary, NC; SAS Institute, Inc.). All results of type I procollagen, type III procollagen, fibronectin, and α-SMA mRNA and Western blots were normalized to control, nonventilated mice breathing room air. ANOVA was used to assess the statistical significance of the differences, followed by multiple comparisons with a Scheffe′s test, and a P value <0.05 was considered statistically significant.

## References

[1] Dreyfuss D, Saumon G (1998). Ventilator-induced lung injury: lessons from experimental studies.. Am J Respir Crit Care Med.

[2] Chaudhary NI, Schnapp A, Park JE (2006). Pharmacologic differentiation of inflammation and fibrosis in the rat bleomycin model.. Am J Respir Crit Care Med.

[3] Matthay MA, Zimmerman GA, Esmon C, Bhattacharya J, Coller B (2003). Future research directions in acute lung injury: summary of a National Heart, Lung, and Blood Institute working group.. Am J Respir Crit Care Med.

[4] Zergeroglu MA, McKenzie MJ, Shanely RA, Van Gammeren D, DeRuisseau KC (2003). Mechanical ventilation-induced oxidative stress in the diaphragm.. J Appl Physiol.

[5] Li LF, Liao SK, Huang CC, Hung MJ, Quinn DA (2008). Serine/threonine kinase-protein kinase B and extracellular signal-regulated kinase regulate ventilator-induced pulmonary fibrosis after bleomycin-induced acute lung injury: a prospective, controlled animal experiment.. Crit Care.

[6] Whidden MA, McClung JM, Falk DJ, Hudson MB, Smuder AJ (2009). Xanthine oxidase contributes to mechanical ventilation-induced diaphragmatic oxidative stress and contractile dysfunction.. J Appl Physiol.

[7] Vassilakopoulos T (2008). Ventilator-induced diaphragm dysfunction: the clinical relevance of animal models.. Intensive Care Med.

[8] Varga J, Rosenbloom J, Jimenez SA (1987). Transforming growth factor beta (TGF beta) causes a persistent increase in steady-state amounts of type I and type III collagen and fibronectin mRNAs in normal human dermal fibroblasts.. Biochem J.

[9] Fahy RJ, Lichtenberger F, McKeegan CB, Nuovo GJ, Marsh CB (2003). The acute respiratory distress syndrome: a role for transforming growth factor-beta 1.. Am J Respir Cell Mol Biol.

[10] Ying S, Shiraishi A, Kao CW, Converse RL, Funderburgh JL (1997). Characterization and expression of the mouse lumican gene.. J Biol Chem.

[11] Dolhnikoff M, Morin J, Roughley PJ, Ludig MS (1998). Expression of lumican in human lungs.. Am J Respir Cell Mol Biol.

[12] Ludwig MS (2007). Proteoglycans and pathophysiology.. J Appl Physiol.

[13] Chakravarti S, Zhang G, Chervoneva I, Roberts L, Birk DE (2006). Collagen fibril assembly during postnatal development and dysfunctional regulation in the lumican-deficient murine cornea.. Dev Dyn.

[14] Hinz B (2007). Formation and function of the myofibroblast during tissue repair.. J Invest Dermatol.

[15] Jubran A (2006). Critical illness and mechanical ventilation: effects on the diaphragm.. Respir Care.

[16] Takala TE, Virtanen P (2000). Biochemical composition of muscle extracellular matrix: the effect of loading.. Scand J Med Sci Sports.

[17] Bai KJ, Spicer AP, Mascarenhas MM, Yu L, Ochoa CD (2005). The role of hyaluronan synthase 3 in ventilator-induced lung injury.. Am J Respir Crit Care Med.

[18] de Carvalho ME, Dolhnikoff M, Meireles SI, Reis LF, Martins MA (2007). Effects of overinflation on procollagen type III expression in experimental acute lung injury.. Crit Care.

[19] Agostini C, Gurrieri C (2006). Chemokine/cytokine cocktail in idiopathic pulmonary fibrosis.. Proc Am Thorac Soc.

[20] Bartram U, Speer CP (2004). The Role of Transforming Growth Factor β in Lung Development and Disease.. Chest.

[21] Pittet JF, Griffiths MJ, Geiser T, Kaminski N, Dalton SL (2001). TGF-beta is a critical mediator of acute lung injury.. J Clin Invest.

[22] McClung JM, Van Gammeren D, Whidden MA, Falk DJ, Kavazis AN (2009). Apocynin attenuates diaphragm oxidative stress and protease activation during prolonged mechanical ventilation.. Crit Care Med.

[23] Moriondo A, Pelosi P, Passi A, Viola M, Marcozzi C (2007). Proteoglycan fragmentation and respiratory mechanics in mechanically ventilated healthy rats.. J Appl Physiol.

[24] Levine S, Nguyen T, Taylor N, Friscia ME, Budak MT (2008). Rapid disuse atrophy of diaphragm fibers in mechanically ventilated humans.. N Engl J Med.

[25] Petrof BJ, Jaber S, Matecki S (2010). Ventilator-induced diaphragmatic dysfunction.. Curr Opin Crit Care.

[26] Al-Jamal R, Ludwig MS (2001). Changes in proteoglycans and lung tissue mechanics during excessive mechanical ventilation in rats.. Am J Physiol Lung Cell Mol Physiol.

[27] ARDSNet (2000). The acute respiratory distress syndrome network. ventilation with lower tidal volumes as compared with traditional tidal volumes for acute lung injury and the acute respiratory distress syndrome.. New Engl J Med.

[28] Matthay MA, Zimmerman GA, Esmon C, Bhattacharya J, Coller B (2003). Future research directions in acute lung injury: summary of a National Heart, Lung, and Blood Institute working group.. Am J Respir Crit Care Med.

[29] Held HD, Boettcher S, Hamann L, Uhlig S (2001). Ventilation-induced chemokine and cytokine release is associated with activation of nuclear factor-kappaB and is blocked by steroids.. Am J Respir Crit Care Med.

[30] Li-Fu Li, Chung-Chi Huang, Yung-Yang Liu, Horng-Chyuan Lin, Kuo-Chin Kao (2011). Hydroxyethyl starch reduces high stretch ventilation-augmented lung injury via vascular endothelial growth factor Trans Res.

[31] Saika S, Shiraishi A, Liu CY, Funderburgh JL, Kao CW (2000). Role of lumican in the corneal epithelium during wound healing.. J Biol Chem.

[32] Kao WW, Funderburgh JL, Xia Y, Liu CY, Conrad GW (2006). Focus on molecules: lumican.. Exp Eye Res.

[33] Li LF, Yu L, Quinn DA (2004). Ventilation-induced neutrophil infiltration depends on c-Jun N-terminal kinase.. Am J Respir Crit Care Med.

[34] Higashiyama H, Yoshimoto D, Kaise T, Matsubara S, Fujiwara M (2007). Inhibition of activin receptor-like kinase 5 attenuates bleomycin-induced pulmonary fibrosis.. Exp Mol Pathol.

[35] Chen J, Kasper M, Heck T, Nakagawa K, Humpert PM (2005). Tissue factor as a link between wounding and tissue repair.. Diabetes.

